# Investigation of the body-centred tetragonal structure of Fe–Co–V–N Bulk foils using the rolling and ammonia-gas-nitriding method

**DOI:** 10.1038/s41598-023-32290-4

**Published:** 2023-04-06

**Authors:** Takashi Hasegawa

**Affiliations:** Department of Materials Science, Akita University, 1-1 Tegata Gakuen-Machi, Akita, 010-8502 Japan

**Keywords:** Magnetic properties and materials, Electrical and electronic engineering, Magnetic materials

## Abstract

Improving the properties of permanent magnets is essential for the advancement of electric motors in reducing energy consumption and carbon emissions. This study investigated the effect of V and N addition to FeCo foils on the stability of tetragonally distorted FeCo-based bulk magnets. The incorporation of these two elements stabilised the body-centred tetragonal structure in thin-film and bulk systems. Fe–Co–V ingots were rolled and nitrided by ammonia gas at 650 °C for 5 h. A body-centred tetragonal lattice with an axial ratio of *c*/*a* ≈ 1.1 was observed by transmission electron microscopy, and the collected data suggested the induction of a large uniaxial magnetic anisotropy. This study is expected to improve our understanding of rare-earth-free magnets.

## Introduction

As electric motors significantly contribute to global electric power consumption, improving their performance is critical for both saving energy and reducing carbon emissions. Permanent magnets are crucial for enhancing the performance of electric motors. The performance of permanent magnets is proportional to the maximum energy product ((*BH*)_max_), which is related to the saturation magnetisation (*M*_s_), remanent magnetisation (*M*_r_), uniaxial magnetic anisotropy constant (*K*_u_), coercivity (*H*_c_), and squareness of the magnetisation curve (*M*_r_*/M*_s_) at the operating temperature, and the Curie temperature (*T*_c_) of the material.

Among commercial permanent magnets, NdFeB has the highest (*BH*)_max_, which approaches the theoretical limit of approximately 500 kJ/m^3^. Therefore, new permanent magnetic materials retaining the advantages of rare-earth-free magnets and outperforming NdFeB are highly sought after.

**Figure 1:**
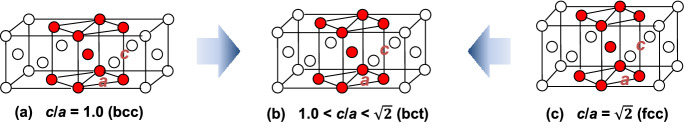
Schematic images of (**a**) bcc, (**b**) bct, and (**c**) fcc structures (based on the Bain relationship typically observed in martensitic materials).

FeCo, which features the highest *M*_s_ among all transition metal alloys, has a body-centred cubic (bcc) structure (Fig. 1a) at room temperature and a high *T*_c_ of approximately 1000 °C, which is three times higher than that of NdFeB. However, bcc FeCo is a soft magnetic material owing to its extremely small *K*_u_.

First-principles calculations predicted a large *K*_u_ of 1.0–10 MJ/m^3^ for FeCo with a body-centred tetragonal (bct) structure (Fig. 1b) and an axial ratio (*c*/*a*) of ~ 1.2^1,2^. To date, the following approaches have been used to synthesise bct FeCo.

(i) Utilisation of the lattice mismatch between the buffer layer and epitaxially grown FeCo.
(ii) Introduction of elements α (substitutional element) and β (interstitial element) to FeCo.

Using method (i), bct FeCo with *c*/*a* ≈ 1.2 was formed by epitaxially growing FeCo films with a thickness (*t*) of < 5 nm on several buffer layers (Pt^3^, CuAu^4^, FePt^5^, and Rh^6,7^). In the thin FeCo films, the *a*-axis was compressed by stress due to lattice mismatch between the FeCo layer and buffer layers. A large *K*_u_ of 1–2 MJ/m^3^ was obtained. A high *H*_c_ of approximately 0.6 T was obtained by micro-fabricating bct FeCo thin films to a grain size of approximately 50 nm using electron beam lithography^7–9^. However, as method (i) requires buffer layers, which induce compressive stress on the *a*-axis of FeCo, method (ii) may be preferable for forming the bct structure in the bulk state. Typically, it is difficult to implement epitaxial growth phenomena in bulk fabrication processes such as melting, rolling, and atomisation.

Before bulk-forming (using method (ii)), FeCo films with added α (such as Al, Ti, and V) and β (such as C and N) elements were epitaxially grown on a Rh buffer layer. In previous studies, this resulted in the successful formation of a bct structure^7,9–13^. Further, a combination of α = V and β = N proved to be the most effective in forming a bct structure with *c*/*a* ≈ 1.2 in thin (*t* ≤ 5 nm) and thick (*t* = 100 nm) films, and a large *K*_u_ of 1 MJ/m^3^ was obtained^9,12–14^. Furthermore, transmission electron microscopy (TEM) imaging of non-epitaxial Fe–Co–V–N films prepared directly on an amorphous SiO_2_ substrate, which does not contribute any beneficial stress to the Fe–Co–V–N layer, revealed the presence of a bct FeCo lattice with *c*/*a* ≈ 1.1^15^.

Herein, we focused on the rolling and ammonia-gas-nitriding method and investigated the effect of N addition on the properties of rolled Fe–Co–V foils to determine the bct FeCo phase in the bulk state.

## Results and discussion

### Crystal structure of Fe–Co–V foils after ammonia-gas-nitriding

**Figure 2:**
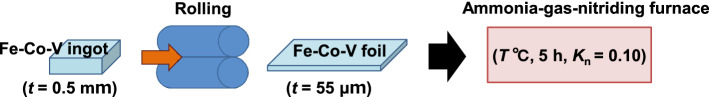
Sample preparation process (rolling and ammonia-gas-nitriding method).

Figure 2 shows the sample preparation process (rolling and ammonia-gas-nitriding method). The Fe–Co–V ingot was melted and rolled into a foil with *t* = 55 µm. The foil was nitrided with ammonia gas at temperatures (*T*) of 550, 600, and 650 °C. The final sample composition measured by X-ray photoelectron spectroscopy (XPS) was (Fe_0.45_Co_0.45_V_0.10_)_100−*x*_N_*x*_ (0 ≤ *x* ≤ 1.5 at.%).

**Figure 3:**
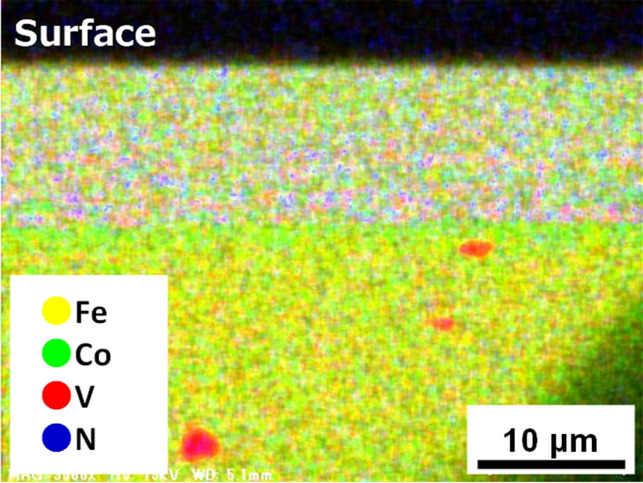
Cross-sectional EDX image of Fe–Co–V–N foil nitrided at *T* = 650 °C.

Figure 3 shows the cross-sectional composition-analysis image of the Fe–Co–V–N foil ammonia-nitrided at *T* = 650 °C and characterised by energy-dispersive X-ray spectroscopy (EDX). In this figure, the yellow (Fe), green (Co), and red (V) spots are randomly mixed, whereas the blue (N) spots are only present in the upper area near the sample surface. Thus, a homogeneous alloy composed of Fe, Co, and V was formed, and N penetrated to a depth of approximately 10 µm from the sample surface. As N penetration proceeded from both the front and back sample surfaces, the volume fraction of the nitrided area was estimated as ~ 36%. At the same time, the presence of red lumps several micrometres in size in the bottom area of Fig. 3 suggests that some V atoms precipitated within this region in the central area of the sample.

**Figure 4:**
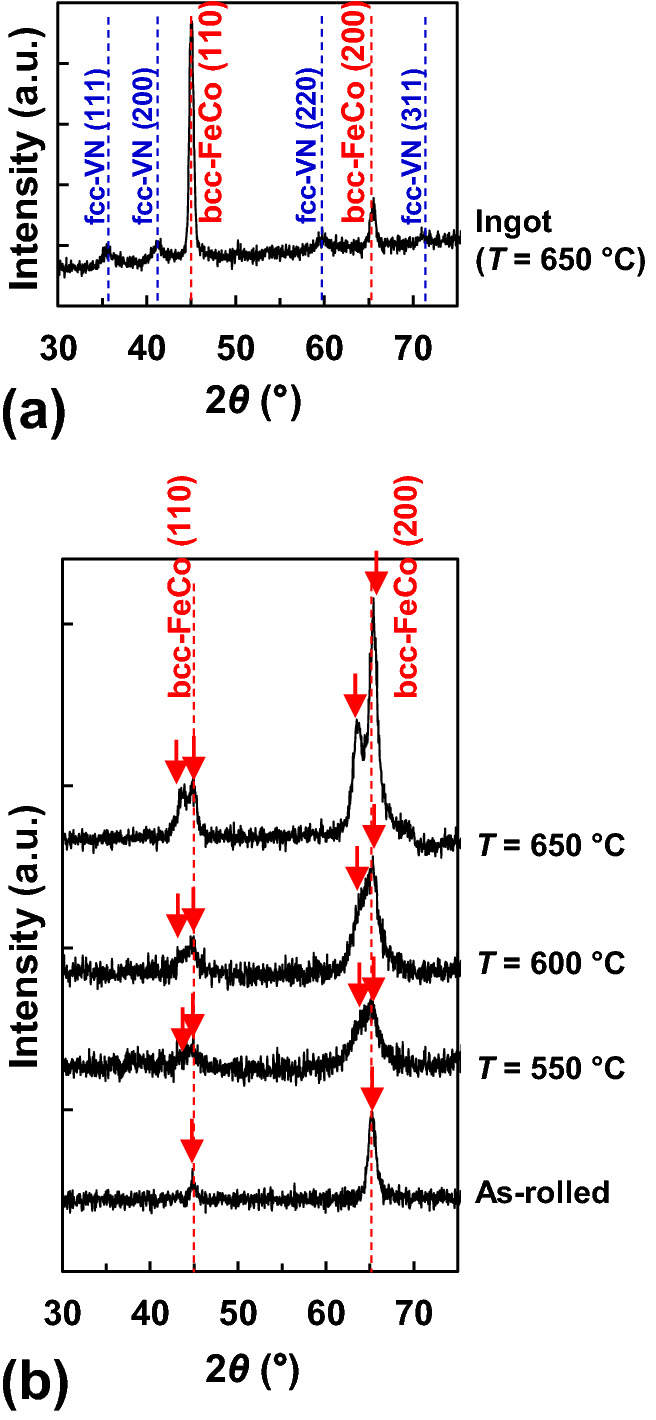
Out-of-plane XRD (*θ*‒2*θ*) patterns of (**a**) Fe–Co–V–N ingot nitrided at *T* = 650 °C and (**b**) Fe–Co–V foil (as-rolled) and Fe–Co–V–N foils nitrided at 550 °C ≤ *T* ≤ 650 °C.

Figure 4 shows the out-of-plane X-ray diffraction (XRD) patterns of samples with and without rolling and ammonia-gas-nitriding. Figure 4a shows the pattern of the non-rolled Fe–Co–V–N ingot (*t* = 0.5 mm) nitrided at 650 °C, revealing that the main peaks corresponded to bcc FeCo (110) (approximately 45°) and bcc FeCo (200) (approximately 65°), respectively. The weaker peaks were ascribed to the face-centred cubic (fcc) V–N structure. These results suggest the formation of a mixed phase containing a fcc V–N structure and a non-oriented polycrystalline bcc FeCo-based alloy.

Figure 4b shows the XRD patterns of the as-rolled Fe–Co–V foil (*t* = 55 µm, without nitriding) and Fe–Co–V–N foils (with rolling, *t* = 55 µm) nitrided at 550, 600, and 650 °C, with red arrows indicating peak positions. The patterns of all samples featured the peaks of bcc FeCo (110) and bcc FeCo (200) at approximately 45° and 65°, respectively. The FeCo (200) peak was more intense than the (110) peak, which suggested a progression of the (200)- and/or (002)-orientation during rolling. Such rolling-induced improvements in the {200}-orientation of FeCo have been reported previously^16^. Other peaks (e.g., fcc V–N) were also observed but were rather weak. Although V-rich phases (red lumps) were observed in the bottom area of Fig. 3, their low volume fractions precluded the detection of the corresponding peaks (typical bcc V (110) and (200) peaks appear at 42.2° and 61.2°, respectively^17^). These results suggest the formation of a polycrystalline FeCo-based alloy with a *c*-axis ([002]-direction) directed parallel and/or perpendicular to the sample plane. The peaks observed at approximately 45° and 65° both changed to twin peaks with increasing *T*. The peak at approximately 45° was split into signals at ~ 43.5° and ~ 45°, and the peak at approximately 65° was split into signals at ~ 63° and ~ 65.5°. Herein, two cases were assumed for the appearance of these twin peaks, with the first one corresponding to the formation of a single-phase Fe–Co–V–N bct structure. Focusing on the twin peak at approximately 65°, the left peak (decreasing angle side) corresponds to the bct FeCo (002) peak, and the right peak (increasing angle side) corresponds to the bct FeCo (200) peak. In this case, the *c*/*a* value of the sample at *T* = 650 °C was estimated as 1.03. The second case assumes the combination of two phases, namely the bcc FeCo phase and the fcc V–N phase. Both cases are discussed in detail in the next section.

### Structural bcc to bct transformation of Fe–Co–V–N foils

**Figure 5:**
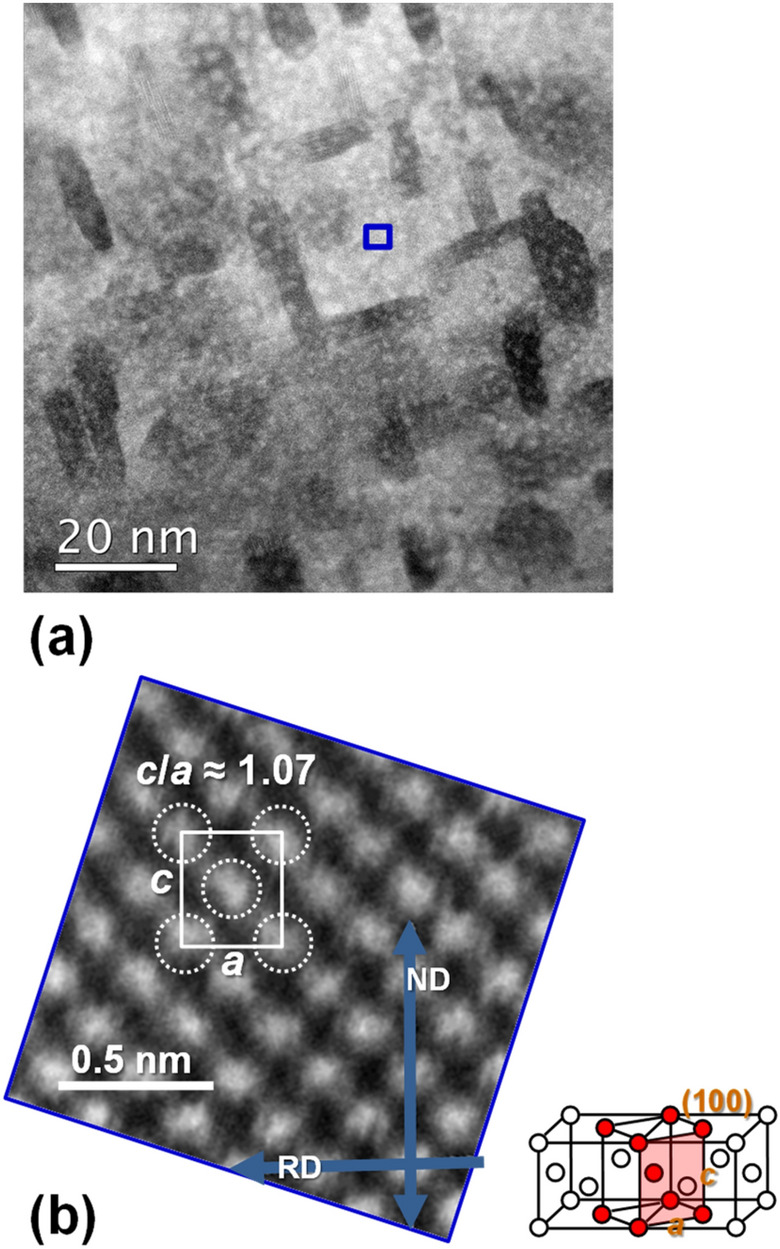
Cross-sectional TEM image of (**a**) Fe–Co–V–N foil nitrided at *T* = 650 °C. (**b**) Enlarged image corresponding to the blue square area–enclosed in (**a**) and showing the rolling direction (RD) and the normal direction (ND). The inset shows the observed (100)-plane.

Figure 5a shows a cross-sectional TEM image of the area near the sample surface (nitrided area) of the Fe–Co–V–N foil nitrided at 650 °C. Dark elongated regions (maximum side length of approximately 20 nm) were observed in the bright matrix and the crystal structure was fcc. The elements detected by TEM–EDX in the dark elongated regions and the bright matrix were Fe, Co, V, and N. Higher V–N contents were detected in the dark areas. Figure 5b shows an enlarged cross-sectional TEM image corresponding to the blue square–enclosed area of Fig. 5a, which has been slightly rotated for easier viewing. In this area, the bright spots correspond to Fe, Co, or V atoms. A bct Fe–Co–V–N lattice with *c*/*a* ≈ 1.07 was observed. Unfortunately, N atoms were not visible in this TEM image, as they appear too light to sufficiently reflect the electron beam but could exist in the *c*-axis as α’’–Fe_16_N_2_^18^.

**Figure 6:**
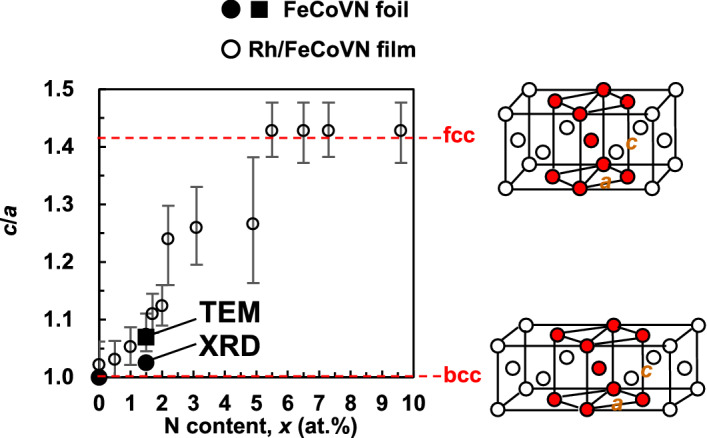
*x*-dependences of *c*/*a* for (Fe_0.45_Co_0.45_V_0.10_)_100−*x*_N_*x*_ foils with *x* = 0 (as-rolled) and 1.5 (nitrided at *T* = 650 °C), and (Fe_0.45_Co_0.45_V_0.10_)_100−*x*_N_*x*_ films epitaxially grown on the Rh buffer layer^12^.

Figure 6 shows the N composition (*x*)-dependences of:

- *c*/*a* (●)—estimated from the XRD twin peaks shown in Fig. 4b,
- *c*/*a* (■) – estimated from the TEM image shown in Fig. 5b, and
- *c*/*a* (○) of the (Fe_0.45_Co_0.45_V_0.10_)_100−*x*_N_*x*_ thin films epitaxially grown on the Rh buffer layer, which has been previously reported by Hasegawa et al.^12^.

The *x*-values of foil samples were determined by analysing the N composition near the sample surface (nitrided area shown in Fig. 3) using XPS. All samples had a similar tendency to transform from a bcc structure to a bct structure with increasing *x*. At *x* = 1.5 at.% of the (Fe_0.45_Co_0.45_V_0.10_)_100−*x*_N_*x*_ foil (nitrided at *T* = 650 °C), the *c*/*a* value estimated from the XRD twin peaks was ~ 1.03, while the *c*/*a* value estimated from the TEM image was ~ 1.07. The latter value (~ 1.07) corresponds to the result obtained for thin films (Rh/FeCoVN). This discrepancy was possibly caused by the difference in the scale of the field of view between XRD and TEM. As mentioned above, this sample was a mix of four phases, namely the bct Fe–Co–V–N phase (which exists in the near-surface region), the fcc Fe–Co–V–N phase with a higher V–N content (which exists in the near-surface region), the bcc Fe–Co–V phase (which exists in the central region), and the V-rich phase (which exists in the central region). XRD measurements provided information on the entire sample, and the reflection peaks represented the average structure. This was due to the spot diameter of the XRD measurements being approximately 1 cm and the X-ray penetration depth being tens of micrometres. In summary, the XRD twin peaks could represent the four phases, and the peaks from the bct Fe–Co–V–N phase with *c*/*a* ≈ 1.07 were obscured possibly due to peak broadening caused by fine crystallisation. In contrast, TEM imaging provided information only on the nitrided local area near the surface and the bct Fe–Co–V–N lattice with the same axial ratio (*c*/*a* ≈ 1.07) as the thin films.

### Magnetic properties of Fe–Co–V–N foils

**Figure 7:**
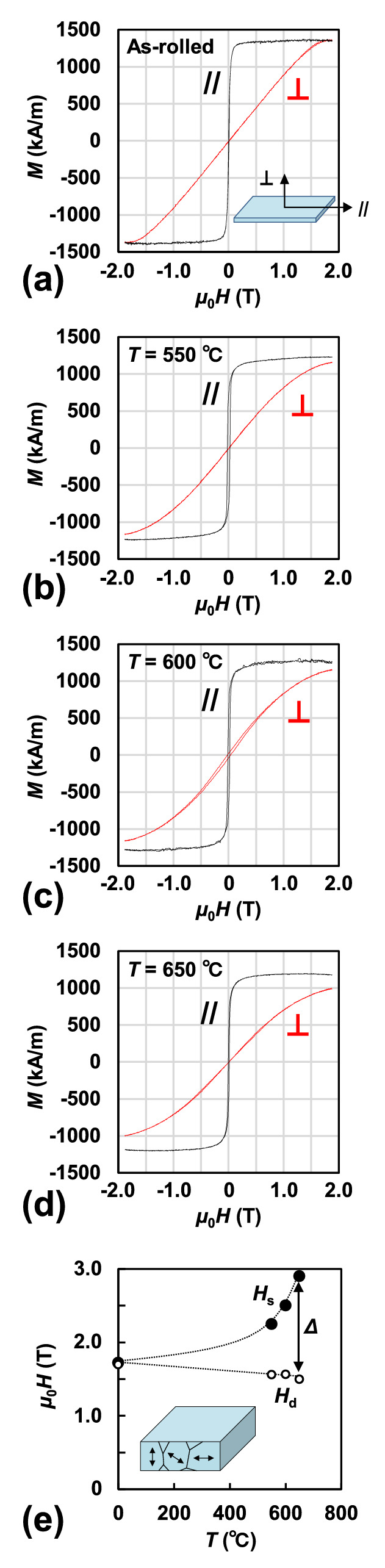
Magnetisation curves of (**a**) Fe–Co–V foil (as-rolled) and Fe–Co–V–N foils nitrided at *T* = (**b**) 550, (**c**) 600, and (**d**) 650 °C. The magnetic field was applied parallel (//) and perpendicular (_┴_) to the sample plane, as shown in the inset of (**a**). (**e**) *H*_s_ and *H*_d_ of Fe–Co–V–N foils plotted as functions of *T*. *Δ* is the difference between *H*_s_ and *H*_d_. The plots at *T* = 0 correspond to the as-rolled sample. The schematic image of the texture is shown in the inset of (**e**).

Figure 7a–d show the magnetisation curves of the as-rolled Fe–Co–V–N foil and foils nitrided at 550, 600, and 650 °C, respectively. The samples were characterised using a vibrating sample magnetometer (VSM). A magnetic field was applied parallel (//) and perpendicular (_┴_) to the sample plane, as shown in the inset of Fig. 7a. As the nitriding temperature increases, the perpendicular magnetisation curves become more difficult to saturate. The magnetic easy axis of the bct Fe–Co–V–N phase is along the* c*-axis^9,12^. The XRD patterns shown in Fig. 4b suggested that the [002]-direction (*c*-axis) was preferentially oriented parallel and/or perpendicular to the sample plane, so the magnetisation curves in Fig. 7b‒d contain the components of both the magnetic easy axis and the hard axis.

In view of the difficulty of evaluating the exact *K*_u_ of non-oriented polycrystalline samples, the intersection points (*H*_s_) between the parallel and perpendicular magnetisation curves were estimated. The *H*_s_-values in Fig. 7b–d were estimated by extrapolating the perpendicular magnetisation curve to the high magnetic field side. *H*_s_ correlated with *K*_u_ and was plotted as a function of *T* (refer to Fig. 7e). The perpendicular demagnetisation field (*H*_d_) was also plotted, as shown in Fig. 7e. *H*_d_ was calculated using the demagnetisation factor (*N*) for the plate shape (*N*_//_ = 0, *N*_┴_ = 1), whose texture was assumed to be continuous magnetically, and the *M*_s_ value.

In Fig. 7e, at *T* = 0 (as-rolled sample), *H*_s_ was almost equal to *H*_d_; therefore, we reasoned that *K*_u_ was small because of the bcc structure. With increasing *T*, *H*_s_ increased, whereas *H*_d_ remained almost constant; subsequently, the difference (*Δ*) between *H*_s_ and *H*_d_ increased. This increase in *Δ* was attributed to the increase in *K*_u_. Usually, in the case of *Δ* = 0, *K*_u_ is zero (except for shape anisotropy); however, in the case of *Δ* ≠ 0, *K*_u_ is non-zero and contains some intrinsic magnetic anisotropy^19^. According to the results, bct Fe–Co–V–N polycrystalline phases having the magnetic easy axis directed parallel and/or perpendicular to the sample plane, and large *K*_u_ formed in samples with *Δ* ≠ 0 nitrided at higher temperatures (e.g., *T* = 650 °C). The origin of the intrinsic anisotropy is not clear on this stage, but it may be connected with the existence of the grains containing the bct Fe–Co–V–N, and the very low *H*_c_ may be caused by the texture with the grains magnetically coupled.

## Summary

The addition of V and N to FeCo foils was investigated for forming stable bct FeCo-based bulk magnets. Foil-shaped Fe–Co–V alloys were prepared by rolling and subsequently nitrided with ammonia gas at high temperatures. For the (Fe_0.45_Co_0.45_V_0.10_)_100−*x*_N_*x*_ (*x* = 1.5 at.%) foil nitrided at *T* = 650 °C, a bct lattice with *c*/*a* ≈ 1.07 was observed by TEM, and the induction of a large *K*_u_ was suggested. The results indicated that the addition of V and N to FeCo stabilised the bct structure in thin-film and bulk states. Therefore, our study facilitates the development of bct FeCo-based permanent magnets.

## Methods

Fe–Co–V–N foils were prepared using the rolling and ammonia-gas-nitriding method shown in Fig. 2. An Fe–Co–V ingot (*t* = 0.5 mm) was prepared by vacuum arc melting from pure elements at temperature above 1500 °C and then rolled into a foil with *t* = 55 µm. The foil was nitrided by ammonia gas with a nitriding potential (*K*_n_ = (*P*_NH3_/*P*_H2_)^3/2^, where *P*_NH3_ and *P*_H2_ are the partial pressures of ammonia and hydrogen gases, respectively) of 0.10 at 550, 600, and 650 °C for 5 h. During nitriding, the flow rates of ammonia and hydrogen gases were 5‒13 and 55‒75 L/min, respectively. The final sample composition was determined by XPS (PHI Model 5600, ULVAC-PHI, Inc.; 300-W Mg *K*_α_ source) as (Fe_0.45_Co_0.45_V_0.10_)_100−*x*_N_*x*_ (0 ≤ *x* ≤ 1.5 at.%). The V content was fixed at 10 at.%, which provided the best balance between *M*_s_ and *K*_u_^14^. EDX coupled with TEM (JEM-ARM200F, JEOL Corp.) was used for local composition analysis. XRD (RINT2000, RIGAKU Corp.) (*θ*‒2*θ* method) with Cu *K*_α_ radiation (40 kV, 40 mA) and TEM were used for crystal structure analysis. A VSM (VSM3S-10, TOEI Corp.) with a maximum field of 2.0 T was used to evaluate magnetic properties, and the corresponding measurements were performed for samples cut into squares with a side of 5 mm.

## Supplementary Information

Supplementary Information.

## Data Availability

All data are available in the supplementary file.
