# Supplementary material for: Investigation of the body-centred tetragonal structure of Fe–Co–V–N Bulk foils using the rolling and ammonia-gas-nitriding method
Source: Sci Rep. 2023 Apr 6;13:5666. doi: 10.1038/s41598-023-32290-4 (PMC10079837; doi:10.1038/s41598-023-32290-4)
Supplement: Supplementary file 1 — Supplementary Information. [file 41598_2023_32290_MOESM1_ESM.docx]

**Supplementary Information**

**Investigation of the body-centred tetragonal structure of Fe-Co-V-N bulk foils using the rolling and ammonia-gas-nitriding method**

Takashi Hasegawa

Department of Materials Science, Akita University, 1-1 Tegata Gakuen-machi, Akita 010-8502, Japan

**Figure S1.** (a) Cross-sectional EDX image for the Fe-Co-V-N foil nitrided at *T* = 650 °C (shown in Fig. 3 in the main text), and (b) compositional-analysis details. Each number in photograph (b) corresponds to the number of the compositional-analysis results. The area near the sample surface (number 10) is clearly nitrided.

**Figure S2.** Cross-sectional TEM images of (a) the area near the sample surface (shown in Fig. 5 in the main text), and (c) the bottom area of Fe-Co-V-N foil nitrided at *T* = 650 °C. (b) and (d) are the enlarged images observed in the blue square area in (a) and (c), respectively. The insets show the observation direction of the (100)-plane. Each number (i, ii) in (b) and (d) corresponds to the number of intensity profiles. The *c*/*a* values estimated from (b) and (d) are 1.07 and 1.00, respectively.

**Figure S3.** Saturation magnetisation of the Fe-Co-V-N foils as a function of *T*. The plot at *T* = 0 correspond to the as-rolled sample.
